# Intestinal Barrier in Post-*Campylobacter jejuni* Irritable Bowel Syndrome

**DOI:** 10.3390/biom13030449

**Published:** 2023-02-28

**Authors:** Sholpan Omarova, Karem Awad, Verena Moos, Christoph Püning, Greta Gölz, Jörg-Dieter Schulzke, Roland Bücker

**Affiliations:** 1 Clinical Physiology, Charité–Universitätsmedizin Berlin, Campus Benjamin Franklin, 12203 Berlin, Germany; 2 Department of Gastroenterology, Infectious Diseases and Rheumatology, Charité–Universitätsmedizin Berlin, Campus Benjamin Franklin, 12203 Berlin, Germany; 3 Department of Veterinary Medicine, Center for Veterinary Public Health, Institute of Food Safety and Food Hygiene, Freie Universität Berlin, 14163 Berlin, Germany

**Keywords:** *Campylobacter jejuni*, irritable bowel syndrome, post-infectious irritable bowel syndrome, barrier function, permeability, antigen entry, leaky gut, tight junction, endocytosis, transcytosis, cytokine

## Abstract

Background: *Campylobacter jejuni* (*C. jejuni*) is one of the most common causes of bacterial gastroenteritis worldwide. One sequela of this infection is the development of post-infectious irritable bowel syndrome (PI-IBS). It has been suggested that a dysfunctional intestinal barrier may promote IBS development. We aimed to test this hypothesis against the background of the leaky gut concept for low-grade inflammation in PI-IBS. Methods: We identified patients with persistent PI-IBS symptoms after *C. jejuni* infection. During sigmoidoscopy, forceps biopsies were obtained for electrophysiological measurements of epithelial transport and barrier function in miniaturized Ussing devices. *C. jejuni* absence was checked by PCR and cytokine production with immunohistochemistry. Results: In PI-IBS, the epithelial resistance of the colon epithelium was unaltered, reflecting an intact paracellular pathway. In contrast, temperature-dependent horseradish peroxidase (HRP, 44 kDa) permeation increased. Short-circuit current (Isc) reflecting active anion secretion and ENaC-dependent electrogenic sodium absorption was unaffected. Early endosome antigen-1 (EEA1) and IL-4 levels increased. *C. jejuni* is not incorporated into the resident microbiota of the colon mucosa in PI-IBS. Conclusions: In PI-IBS after *C. jejuni* infection, macromolecule uptake via endocytosis was enhanced, leading to low-grade inflammation with pro-inflammatory cytokine release. The findings will allow *C. jejuni*-induced pathomechanisms to be targeted during infection and, thereafter to reduce sequelae such as PI-IBS.

## 1. Introduction

*Campylobacter* enteritis can lead to sequelae such as Guillain–Barré syndrome or reactive arthritis. Another complication after an infection with *Campylobacter jejuni* (*C. jejuni*) is the development of irritable bowel syndrome (IBS), first described by Spiller and colleagues in 2000 and associated with increased activation of enteroendocrine cells (EC) [1]. As a pathomechanism, it has been established that the release of serotonin from EC cells causes a dysregulation of intestinal motility and visceral hypersensitivity in post-infectious IBS (PI-IBS) [1]. Since *C. jejuni* infection is one of the most common bacterial enteritis worldwide, the control of this infection and its sequelae is an urgent public health concern [2]. IBS has a prevalence of 10–20% [3] and PI-IBS is observed in approximately 10% of patients after *C. jejuni* infection [4]. In PI-IBS, it is difficult to identify inherent pathomechanisms, such as those responsible for diarrhea or constipation due to the mixing of the different IBS subtypes, such as diarrhea-predominant IBS (IBS-D) and constipation-predominant IBS (IBS-C). In acute campylobacteriosis, the malabsorption of sodium and epithelial barrier dysfunction of ions leading to leak-flux diarrhea have been identified as the predominant diarrheal mechanisms in the colon [5]. The entry of the bacterial luminal antigen, such as lipooligosaccharides (LOS), into the mucosa proved to be the most intensively induced pathway in RNA-sequencing-based signaling pathway analysis [5]. This is considered to induce mucosal inflammation, which in turn impairs intestinal barrier function via the release of cytokines, leading to tight junction (TJ) deregulation and the induction of epithelial leaks [5]: a vicious circle.

Natural compounds have been identified that may combat *C. jejuni* infection by enhancing barrier function and down-regulating immune activation, namely the polyphenols resveratrol and curcumin, and the pre-hormone vitamin D [6,7,8]. Complementary intervention strategies of this type are conceivable for acute infections, but could also be useful for oral use in the recovery phase of an infection or afterwards for the prevention of PI-IBS. In our present study, we performed a follow-up of acutely infected *C. jejuni* patients treated in the Charité–Universitätsmedizin, Berlin, who presented with long-term complications of PI-IBS. Our aim was to electrophysiologically characterize the epithelial barrier function of colon biopsies from patients using Ussing chambers, including macromolecule permeability measurements and an analysis of TJ integrity to detect a possible leak pathway or accelerated antigen influx (leaky gut phenomenon).

## 2. Materials and Methods

### 2.1. Study Subjects

In this study, 7 patients with PI-IBS were recruited between 2018 and 2020 at the Charité–Universitätsmedizin, Berlin. These patients met Rome IV criteria for the diagnosis of IBS at least 12 months after an acute episode of *C. jejuni* enteritis. Other gastrointestinal disorders, such as microscopic colitis or inflammatory bowel disease (IBD), were ruled out using upper endoscopy, colonoscopy and MR enterography. Pregnant women and individuals with self-reported lactose intolerance, food allergy and psychosocial factors affecting the digestive system were excluded. During colonoscopy biopsies were obtained from the sigmoid colon.

Healthy patients who underwent screening colonoscopy were recruited as controls (n = 7). None had gastrointestinal symptoms or evidence of intestinal disease at colonoscopy or a medical history of gastrointestinal disease. With this small sample size of n = 7 per group we made our measurements and conducted analyses.

### 2.2. Ethics

This study adhered to the Declaration of Helsinki for the use of human intestinal biopsies, blood samples and questionnaires. For the project “Investigation on barrier function in post-infectious irritable bowel syndrome”, ethical approval was obtained from the institutional review board “Ethics Committee of the Charité” under the approval number, EA4/025/11. Written informed consent was obtained from each patient.

### 2.3. Campylobacter-Specific PCR from Stool and Mucosal Samples

In order to determine the presence or absence of *C. jejuni* in stool or mucosal tissue of patients, DNA was extracted and further analyzed using multiplex qPCR. For the extraction of DNA from stool samples, the QIAamp DNA stool mini kit (Qiagen, Hilden, Germany) was used, following the manufacturer’s instructions for the protocol to isolate DNA from stool for pathogen detection. DNA extraction from mucosal samples was carried out using the DNeasy Blood & Tissue Kit (Qiagen) and following the protocol for the purification of total DNA from animal tissues, according to the manufacturer’s instructions. Following extraction, a qPCR was performed, based on *C*. *jejuni*-specific mapA gene, using primers and probe described by [9]. Briefly, each reaction had a volume of 25 µL, containing 1 × Maxima Probe/ROX qPCR Master Mix (Thermo Scientific, Schwerte, Germany), 375 nM of each primer, 100 nM of probe labeled with FAM and quencher BHQ-2 (all Metabion, Planegg, Germany), 5 µL DNA template as well as 5.5 µL sterile water. The qPCR was performed using a CFX96 Real-Time Detection System (Bio-Rad, Hercules, CA, USA) with amplification parameters of 50 °C for 2 min and 95 °C for 10 min, as well as 45 cycles of 95 °C for 15 s, 60 °C for 30 s and 72 °C for 30 s. Each sample was run in a technical duplicate, with positive and negative samples for comparison.

### 2.4. Electrophysiological Measurements on Colon Biopsies

**Impedance spectroscopy**. Colon biopsies were mounted in miniaturized Ussing chambers (0.049 cm^2^ area). Total transepithelial electrical resistance (R^t^ = TER) of the colon mucosa consists of two components, the epithelial resistance (R^epi^) and subepithelial resistance (R^sub^), which can be differentiated using impedance spectroscopy [10]. Alternating currents (ranging from 1 Hz to 65 kHz) were applied using a programmable frequency response analyzer (402, Beran Instruments, Devon, UK) and an electrochemical interface (1286, Solartron Schlumberger, Farnborough, UK). From the voltage responses, the complex impedance was obtained. The electrical resistance of the bath was used for correction. Each impedance locus plot is depicted in a Nyquist diagram. For the interpretation of impedance plots, a 3-parameter model was sufficient, in which the epithelium is given as an RC unit of a resistor (R^epi^) in parallel to a capacitor (C^epi^), and the subepithelial tissue layers as a resistor (R^sub^) in series to this RC unit. Total transepithelial resistance (R^t^) was obtained from the intersection of the semicircle with the abscissa at frequency 0 and subepithelial resistance (R^sub^) from the intersection of the semicircle, with the abscissa at an infinite frequency. Epithelial resistance (R^epi^) was obtained from R^t^ minus R^sub^.

**Electrogenic sodium absorption**. In parallel Ussing chamber experiments, human biopsies were stimulated with aldosterone (3·10^−9^ M). Eight hours after stimulation, the activity of the epithelial sodium channel (ENaC) was determined by addition of the ENaC blocker amiloride (10^−4^ M) to the mucosal compartment and the resulting drop in I_SC_ was assigned to an ENaC-dependent sodium absorption, as previously described [11].

**Electrogenic anion secretion**. In parallel Ussing experiments, the short-circuit current (I_SC_) of human colon biopsies was measured in order to quantify basal rheogenic anion secretion. At the end of each experiment, Isc was simulated via the addition of theophylline (10^−2^ M, Sigma-Aldrich, St. Louis, MO, USA) and prostaglandin E_2_ (PGE_2_, 10^−6^ M, Sigma-Aldrich, St. Louis, MO, USA) for the induction of Cl^-^ secretion via cAMP, followed by the muscarinic receptor agonist carbachol (10^−4^ M, Sigma-Aldrich, St. Louis, MO, USA) to determine the maximal secretory capacity of the epithelium, in order to test the viability of the tissue under in vitro conditions.

**Permeability measurements**. Unidirectional flux measurements with 100 µM fluorescein (332 Da; Sigma-Aldrich, St. Louis, MO, USA), 300 µM dialyzed fluorescein isothiocyanate (FITC)-dextran-4000 (4 kDa; TdB Consultancy, Uppsala, Sweden) or 18 µM horseradish peroxidase (HRP, 44 kDa, Sigma-Aldrich, St. Louis, MO, USA) were performed in patients’ biopsies from the mucosal to serosal direction in Ussing chambers under voltage–clamp conditions, as previously described [12]. At 20 min intervals, samples were taken from the basolateral hemichamber. Fluorescence or enzymatic activity was measured using a spectrofluorimeter (Tecan, Maennedorf, Switzerland), and permeability was calculated from flux over concentration difference. For transcellular antigen passage, 44 kDa HRP was used as a marker molecule. To distinguish between transcytosis and paracellular leakage, HRP fluxes were measured at different temperatures: (i) at body temperature, which allows for transcytotic transport and paracellular passage; and (ii) at 12 °C, when transcytosis is inactive and only paracellular passage is ongoing. Lowering the temperature inhibits active endosomal transport, while the passive paracellular transport pathway remains largely unaffected [13]. In this study, however, we lowered the temperature only to 12 °C, and not to 4 °C as in previous studies [14], since changes in the TJs can also occur at low temperatures. This assessment is based on the fact that no TJs could be formed in the intestinal cell line HT-29 at temperatures below 10 °C [15]. It is therefore most likely that by cooling the cells down to only 12 °C in our experimental design, not all active processes in the cells are inhibited, and an increase in HRP permeability is still detectable in the cooled cells. In cardiac muscle cells, it has already been shown that lowering the temperature partially, but not completely, inhibits HRP transport [16].

### 2.5. Immunohistochemistry of Colonic Epithelium

Immunostaining on paraffin sections of colonic biopsies was performed as previously described [17]. Briefly, 3 µm paraffin sections were deparaffinized followed by retrieval of the antigenic epitopes via treatment in a pressure cooker in citrate buffer pH 6 for 3 min. Unspecific bindings were blocked by a serum-free protein block (DAKO, Glosdrup, Denmark) for 15 min at room temperature and the following polyclonal primary antibodies—rabbit-anti-human-EEA1 (abcam, Cambridge, UK), IL-1β (Bioss, Woburn, MA, USA), IL-22 (abcam), IL-4 (Tebubio, Frankfurt, Germany), IL-10 and IFNγ (both Peprotech, Hamburg, Germany), and goat-anti-human-IL-6 (R&D systems, Minneapolis, MN, USA) and -TNFα (PeproTech)—were applied in dilutions previously titrated in antibody diluent (DAKO) at 4 °C overnight. Stainings were visualized using donkey-anti-goat or donkey-anti-rabbit Biotin (Dianova, Hamburg, Germany), followed by the use of Streptavidin-alkaline phosphatase (DAKO) (both 1 h at room temperature) and ImmPACT VectorRed substrate Kit (Vector laboratories, Newark, CA, USA). Nuclei were counterstained with hematoxylin, and washing between the single steps was performed with TBS 0.1% Tween (DAKO). Intensity gradings of EEA1- or cytokine-positive enterocytes were determined using 6 biopsies per group, each of which was obtained as the result of analyzing epithelial cells within 5 high-power fields of 0.237 mm^2^ each. Instead of counting the percentage of cells with an active staining as used for characterizing the activation of immune cells, we semi-quantitatively evaluated the intensity of cytokine staining of epithelial cells as readout cytokine action on the epithelium.

### 2.6. Statistics

Data are presented as individual values and means ± standard deviations (SD), and a statistical comparison with Student’s *t*-test was performed using GraphPad Prism (version 7.0, GraphPad Software Inc., San Diego, CA, USA). *p* < 0.05 was considered statistically significant.

## 3. Results

### 3.1. Patients’ Disease Characteristics

Post-infectious IBS patients were identified by following up patients after an episode of *C. jejuni* infection. Patients presenting with episodes of diarrhea and/or obstipation at least one year after *C. jejuni* infection were assessed for IBS using Rome IV criteria. Seven patients were included, five of the diarrheal type (IBS-D), one of the constipation type (IBS-C), and one unclassified type (IBS-U). Then, at the time of colonoscopy, a PCR test for *C. jejuni* was performed. All seven patients had a negative test result, which speaks against inclusion in the patient’s microbiome.

### 3.2. Active Sodium Absorption through the Epithelial Sodium Channel (ENaC) Was Not Affected

**Figure 1:**
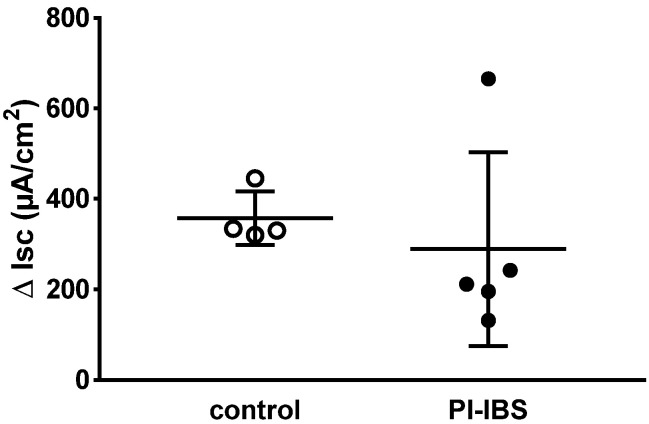
Electrogenic sodium transport measured in miniaturized Ussing chambers. Sodium transport J_Na_ was determined after aldosterone stimulation as the decrease in short-circuit current Isc (ΔIsc) after adding amiloride, a selective ENaC channel blocker. ENaC-dependent electrogenic sodium transport was measured in colon biopsies of four healthy (control) and five post-infectious irritable bowel syndrome (PI-IBS) patients (no significant difference between the groups in Student’s *t*-test).

In biopsies of PI-IBS patients, epithelial sodium channel (ENaC)-dependent active sodium absorption was measured as amiloride-sensitive short-circuit current and was found to be unchanged compared to the control (Figure 1). In particular, this active transport was investigated because its activity was severely impaired in acute campylobacteriosis [5]. Thus, ENaC-dependent sodium malabsorption as a diarrhea mechanism in PI-IBS can be ruled out.

### 3.3. Electrogenic Anion Secretion

**Figure 2:**
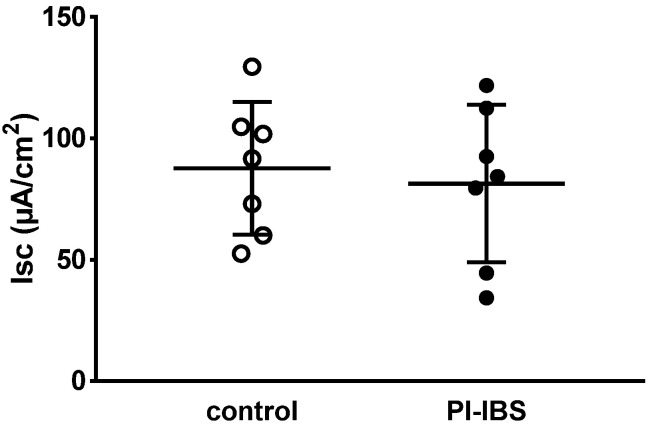
Basal active electrogenic anion secretion was characterized by measuring the spontaneous short-circuit current (Isc) of sigmoid colon mucosae from patients with post-infectious irritable bowel syndrome (PI-IBS) and their respective controls (n = 7 each; n.s.).

Decreased active anion secretion could explain constipation, while increased active anion secretion favored diarrhea, but the data in PI-IBS were unchanged, disproving such a pathophysiology (Figure 2).

After maximal stimulation of electrogenic ion transport with PGE_2_, theophylline and carbachol, no significant difference could be detected compared to the control (ΔIsc = 201 ± 132 µA/cm^2^ in PI-IBS versus 160 ± 132 µA/cm^2^ in controls, n = 5–7 each, *p* = 0.54). Thus, viability of the mucosal samples from the PI-IBS patients was not compromised during the course of the in vitro experiment.

### 3.4. Impedance Spectroscopy for Determining Epithelial Resistance (R^epi^)

**Figure 3:**
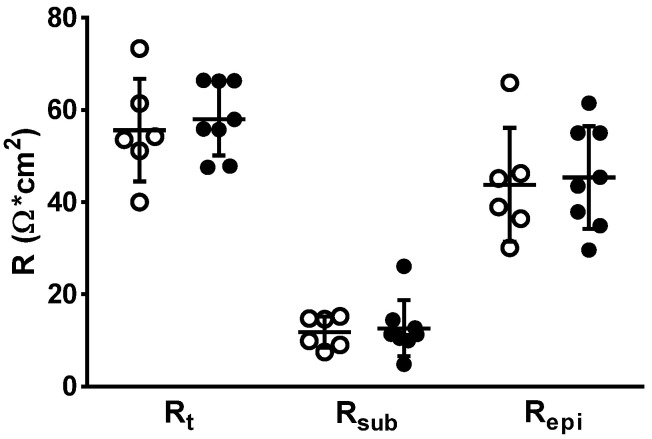
In impedance spectroscopy, epithelial (R^epi^) and subepithelial resistance (R^sub^) contributions to the overall electrical resistance (R^t^) in human colon biopsies. R^sub^ reflects differences in subepithelial tissue layers, e.g., as a result of inflammation. In case of an inflamed subepithelium, the increased R^sub^ may mask the overall resistance defect (R^t^). White circle = control values, black circles = PI-IBS values. No differences were found between groups using Student’s *t*-test.

Colon mucosae were examined for changes in transepithelial (transmural) electrical resistance (R^t^). Impedance spectroscopy was applied to distinguish subepithelial resistance (R^sub^) from epithelial resistance (R^epi^). However, no change was observed between both groups, neither in R^t^_,_ R^epi^ nor R^sub^, confirming the absence of high-grade inflammatory processes in the mucosa and submucosa of PI-IBS patients (Figure 3). This is also direct evidence for an intact epithelial barrier function for ions of the PI-IBS mucosa.

### 3.5. HRP Fluxes Indicate Macromolecule Permeability

**Figure 4:**
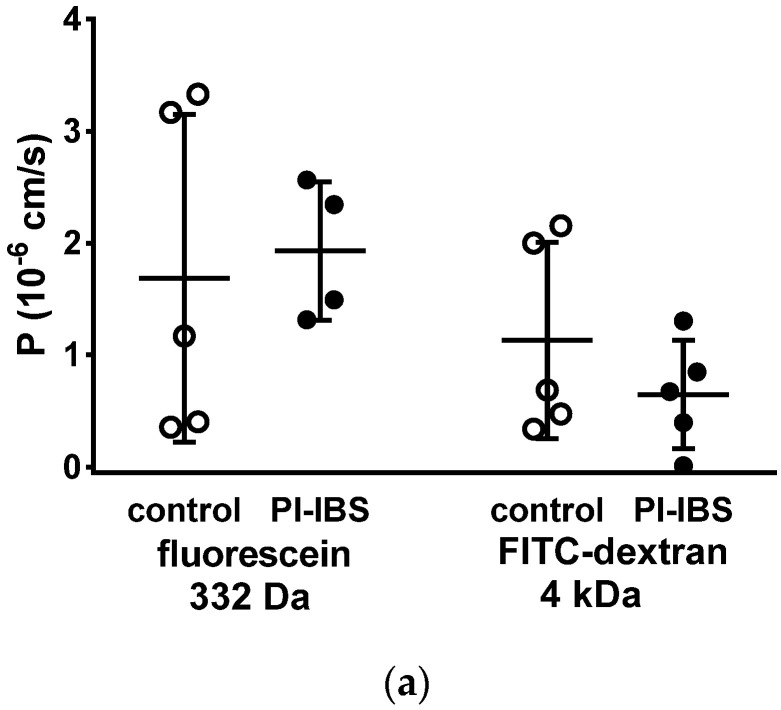

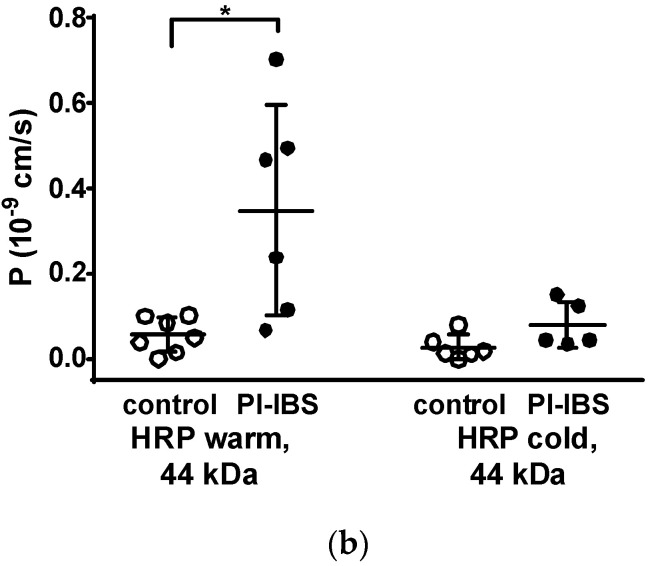
Macromolecule permeability in the sigmoid colon. Tracer fluxes across intestinal biopsy tissues were determined in Ussing chambers. Marker molecule fluxes of different sizes were measured in mucosal-to-serosal direction with (**a**) 332 Da fluorescein and 4 kDa FITC-dextran, and (**b**) horseradish peroxidase (HRP, 44 kDa), either in warm conditions (37 °C, body temperature) or cold conditions (12 °C, causing endocytosis arrest). * *p* < 0.05, Student’s *t*-test, n = 6–7.

To assess barrier function beyond ion permeability (pore pathway), we conducted tracer flux measurements with marker molecules of different sizes and passage routes. All of these transport measurements were performed in Ussing chambers in a mucosal to serosal direction. In mucosae from PI-IBS patients, permeability was unaltered for 332 Da fluorescein (9 Å) as well as for 4 kDa dextran (14 Å) (Figure 4a). This was studied to characterize the leakiness of TJs, which finally would facilitate the passage through the paracellular pathway (leak pathway).

However, the larger protein, horseradish peroxidase, (HRP, molecular weight 44 kDa) when measured at 12 °C, acts as a marker molecule for the unrestricted pathway with passage characteristics > 100 Å, which could be caused by leakage through damaged tricellular TJ or unrepaired cell loss in the epithelium. Moreover, HRP can also be used as a marker for transcytotic protein uptake via enterocytes, namely when the difference between transport rates at 37 °C and at 12 °C is considered. In our present study, HRP macromolecule permeation was found to be increased in PI-IBS patients at 37 °C but not at 12 °C (Figure 4b). This points to an increase in transcellular HRP uptake via transcytosis in PI-IBS patients, while paracellular uptake was unaffected.

### 3.6. EEA1 as a Marker for Endocytosis

**Figure 5:**
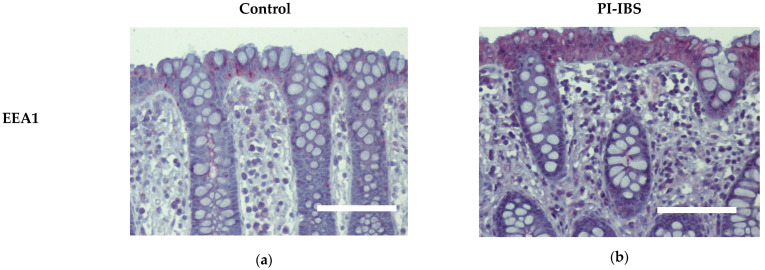
Immunohistochemistry of early endosome antigen-1 (EEA1) at surface enterocytes in colon mucosa of (**a**) healthy controls and (**b**) PI-IBS patients. Red: EEA1; violet: nuclei counterstaining with hematoxylin. Bar = 100 µm.

Biopsies from the sigmoid colon were fixed with formaldehyde, sectioned and stained for early endosome antigen-1 (EEA1). Labelling with antibodies against EEA1 revealed an apparent enrichment of EEA1 signals within colon surface enterocytes of IBS patients, which is compatible with ongoing endocytosis (Figure 5, Table 1).

### 3.7. Cytokine Signals of IL-1β, IL-4 and IL-22 Are Intensified on Colon Epithelial Cells

**Figure 6:**
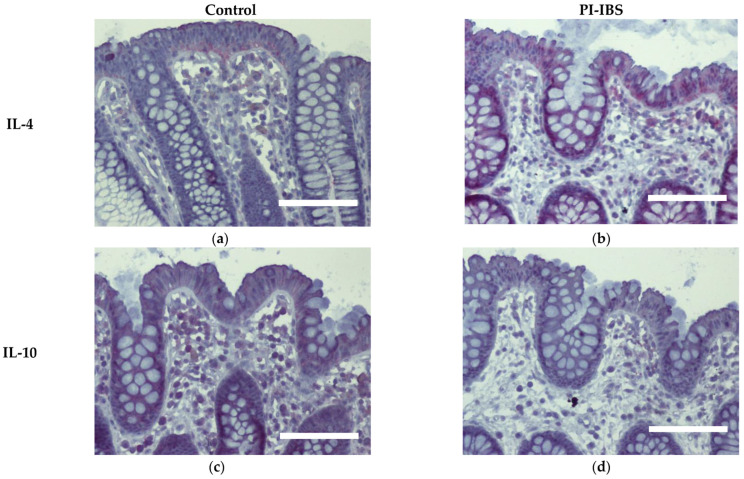
Immunohistochemistry of the Th2 cytokines IL-4 and IL-10 (red) in the colon mucosa of controls (**a**,**c**) and PI-IBS patients (**b**,**d**) (counterstaining with hematoxylin = violet). Staining for IL-4 intensified in surface enterocytes in PI-IBS. Bar = 100 µm.

**Figure 7:**
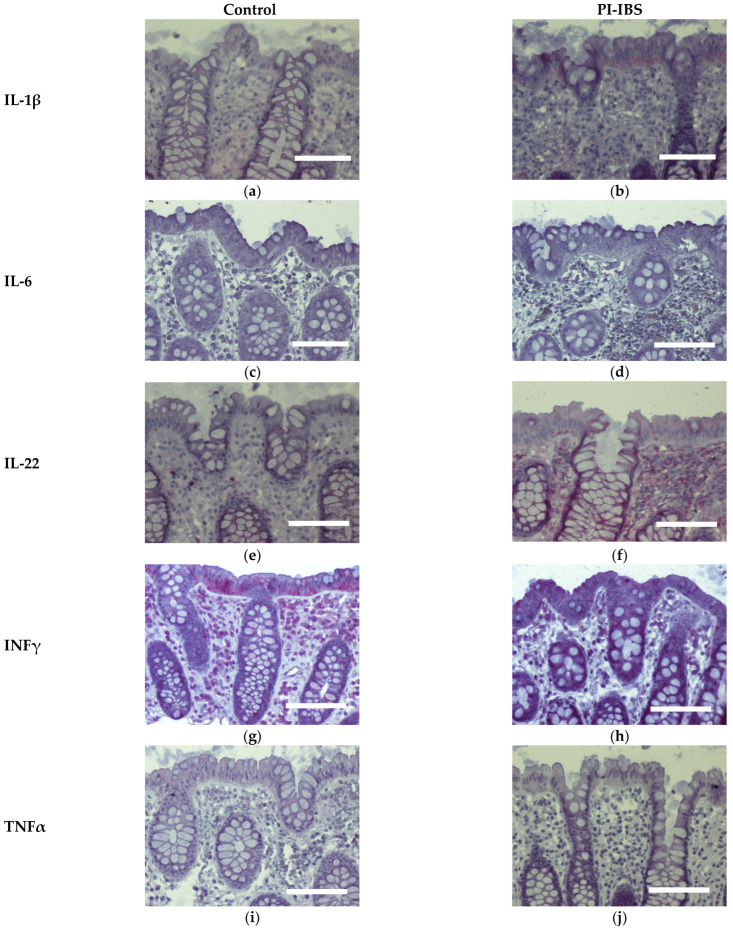
Immunohistochemistry of Th1 cytokines (red) in the colon mucosa of controls and PI-IBS patients of IL-1β (**a**,**b**), IL-6 (**c**,**d**), IL-22 (**e**,**f**), INFγ (**g**,**h**) and TNFα (**i**,**j**), (counterstaining with hematoxylin = violet). Staining for IL-1β and IL-22 intensified in surface enterocytes in PI-IBS. Bar = 100 µm.

Biopsies from the sigmoid colon were also stained for the anti-inflammatory cytokines IL-4 and IL-10 (Figure 6) or the pro-inflammatory cytokines IL-1β, IL-6, IL-22, INFγ and TNFα (Figure 7). In parallel series of stainings, the Th2 cytokine IL-4 was found to be intensified at surface enterocytes in PI-IBS, whereas IL-10 remained unchanged (Figure 6, Table 1). In accordance with the pathophysiological view of a low-grade Th1 inflammatory response in some IBS patients, we found a more intense staining of IL-1β at surface enterocytes, but not with the other pro-inflammatory cytokines: IL-6, TNFα and IFNγ (Figure 7, Table 1). Surprisingly, the pro-inflammatory cytokine IL-22 also intensified in surface enterocytes in our PI-IBS cohort (Figure 7e,f, Table 1).

## 4. Discussion

### 4.1. Characterization of Epithelial Barrier Function in the Colon

Intestinal barrier function is the complex result of the interaction of the microbiota, mucus, epithelial cell layer with its cell–cell junctions, and the innate immune system within the mucosa. Ion permeability is mainly determined using the epithelial TJ, in which transmembrane proteins seal the paracellular space from the intestinal lumen. So far, 26 different claudins have been identified that regulate ion and water permeability of the human intestine, mostly as barrier-forming claudins. However, some of them are also channel builders, such as claudin-2 and -15. The latter type of TJ proteins passively allow ions and water to pass through the epithelium and in this way can contribute to diarrhea. There is another group of sealing TJ proteins, the tight junction-associated marvel proteins (TAMPs) occludin and tricellulin, that are relevant for sealing the tricellular TJ, which is a natural weak point for the passage of macromolecules across the epithelium [18,19]. Finally, transepithelial antigen uptake by transcytosis is also possible, which means that even large macromolecules can be absorbed into the mucosa.

While intestinal barrier function for ions can be easily analyzed using electrical resistance measurements in Ussing chambers, the barrier function against macromolecules passing through the tricellular TJ or smaller macromolecules passing through the bicellular TJ of the intestinal epithelium can only be characterized using flux measurements, namely via suitable molecular tracers of different sizes, such as fluorescein (332 Da) or different FITC dextrans, usually in the range between 1 and 20 kDa. However, neither resistance measurements of R^epi^ via impedance spectroscopy nor flux studies showed any change in barrier function in our PI-IBS patients. However, it turned out that another mechanism of antigen uptake was altered in PI-IBS, namely transcytotic protein uptake.

As the most important finding for barrier pathology in our present study, an increase in macromolecule passage, measured as a temperature-dependent increase in HRP uptake was found in our PI-IBS cohort. In the functional measurements on the colon mucosa of PI-IBS patients, the transcytotic pathway was activated, as demonstrated by an increased HRP flux at 37 °C but not at 12 °C. This uptake of macromolecules reflects the transcytosis of antigens from the intestinal lumen into the mucosa, which has important pathological consequences for mucosal homoeostasis when accelerated. This type of permeability measurement has two advantages over conventional unidirectional tracer flux measurements with FITC-labeled dextrans. By detecting its enzymatic activity, HRP flux quantification principally rules out that smaller cleavage products of this tracer are misinterpreted (avoiding overestimation of fluorescence uptake rates in, e.g., FITC-dextrans). Furthermore, temperature sensitivity can discriminate active transcellular uptake from passive penetration via huge paracellular leaks (the unrestricted pathway). In this manner, the increase in active transcytotic HRP uptake in our present study is important evidence for increased endocytotic antigen uptake in PI-IBS.

### 4.2. Diarrhea and/or Constipation in IBS

Since episodes of diarrhea and constipation in IBS patients may be caused by changes in the activity of intestinal ion transport processes, the regulatory influence on rheogenic anion secretion is an important consideration. Furthermore, transporters involved in absorption of Na^+^ and Cl^−^, such as the ENaC (epithelial sodium channel), NHE3 (sodium-hydrogen antiporter 3) or DRA (down-regulated in adenoma), became the focus of our attention since they can be influenced by pro-inflammatory cytokines, as also shown in campylobacteriosis [5]. Indeed, it seems plausible to conclude that active ion transport might be up- or down-regulated during the course of infectious enteritis, such as that caused by *C. jejuni*, which is then carried forward by low-grade mucosal inflammation, secondary to this epithelial barrier disorder in PI-IBS.

However, such changes in epithelial ion transporter activity were not detected in our PI-IBS cohort. Although Isc measurements can only rule out electrogenic rather than electroneutral transport processes, it is relatively likely that the lack of significant Isc changes was due to the heterogeneity of our PI-IBS group, which includes both IBS-D, IBS-C and IBS-U patients. Another explanation for changes in stool consistency without altered anion transport in PI-IBS patients could be intestinal motility disorders, which are another hallmark of IBS and can contribute to both constipation and diarrhea. The predominant symptom in some of our PI-IBS patients was loose stools. This could also indicate a shortening of the contact time of ingesta with the absorptive surface area and may co-occur with visceral hypersensitivity due to overreactive intrinsic primary afferent neurons (IPANs) [20].

Changes in TJ proteins toward more leakiness would provide an additional explanation for loose stools in PI-IBS patients, even if contact time remained unchanged. It should be noted, that this is the first set of paracellular ion permeability measurements in PI-IBS. However, as mentioned above, no such barrier dysfunction in the leak pathway has been previously reported in PI-IBS patients. Therefore, our findings may help to understand why these PI-IBS patients are often IBS-C or IBS-M patients [1].

### 4.3. Leaky Gut Concept and Cytokine Induction of Barrier Disturbance

Interestingly, it has been shown that the activity of transcytosis processes in enterocytes can be modulated by cytokines [1], which could make this mechanism the missing link in the vicious circle of the leaky gut. Beyond the acute phase of an infectious gastrointestinal disease with a pronounced barrier defect, it is assumed that low-grade inflammatory reactions in the intestinal mucosa can persist during the development of IBS [21] and that this continuous antigen uptake into the mucosa could be the trigger of persistent barrier dysfunction. Additionally, it is quite conceivable that such a persistent barrier defect can be caused by transcytotic antigen uptake.

Another explanation would be bacterial invasion, such as by *C. jejuni*. All bacterial proteins from the lumen can be considered potential antigens, with the most potent molecule family for immune activation being bacterial antigens such as lipooligosaccharides (LOS). This entry of antigens from the lumen into the mucosa, whether via bacterial invasion or transcytosis of bacterial proteins, can lead to immune activation and self-permissive barrier dysfunction through the release of pro-inflammatory cytokines, termed the leaky gut concept [22]. Features of the leaky gut concept have been described for inflammatory bowel diseases and infectious gastroenteritis, e.g., in ulcerative colitis and for the generation of focal leaks in the colonic mucosa by B2 *Escherichia coli* [12].

While transcytotic antigen uptake via M-cells predominates in the ileum, it is primarily absorptive enterocytes in the small and large intestine that are capable of endocytotic antigen uptake. Normally, after endocytotic uptake, antigens are then intracellularly degraded in lysosomes, and only a small amount of unprocessed protein enters the mucosa via the basolateral membrane [23]. However, this undegraded part can be relatively significant in epithelial inflammatory responses, especially in celiac disease, where a high percentage of gliadins remain intact during transcytosis and reach immune cells in the subepithelium [24]. In this case, a significant amount of macromolecules is taken up and can trigger a pronounced cytokine response [23,25].

In our study, macromolecule uptake was indeed increased as the endocytosis marker EEA1 was immunohistochemically observed to be enhanced in enterocytes from PI-IBS patients, confirming the activation of the endocytic uptake pathway for antigens. In addition, the immunohistochemistry of mucosal samples revealed that cytokines IL-4, IL-1β and IL-22 were induced in epithelial cells of the PI-IBS group, supporting the notion of stimulation of transcytotic antigen uptake in PI-IBS. Other cytokines were not induced, which is consistent with the literature, where most measurements in blood serum and intestinal samples were negative for potential inflammatory markers. However, some studies have demonstrated a higher cytokine production, such as for IL-1β in colorectal biopsies from IBS patients [26,27]. The same is true in our patients’ mucosal samples, which showed an activation of IL-1β and no other pro-inflammatory cytokines, as far as we examined for central inflammatory cytokine pathways, such as IL-6 and TNFα. Surprisingly, we found the activation of the Th2 cytokine IL-4 (and no inhibition of IL-10). Consistent with this, in another study on an IBS-M cohort, we found IL-4 is an upstream regulator, calculated as a bioinformatics prediction in an *ingenuity pathway analysis* (IPA) from RNA-seq data [28]. Furthermore, *C. jejuni* was shown to induce IL-4 production in the mouse colon 5 weeks after infection [29]. Moreover, when IL-4 was added to porcine intestinal epithelial cells (IPEC-1), endocytotic activity increased, resulting in an uptake of *C. jejuni* and *E. coli* [30]. Additionally, in T84 cells, IL-4 has been shown to induce transcytosis as evidenced by increased HRP fluxes [13,14]. This hyperactivation of transcytosis in epithelial cells was accompanied by the observation of hyperactive EC cells in PI-IBS after *C. jejuni* infection [1]. However, the type of endocytosis stimulated in PI-IBS needs to be further investigated in the future. Nevertheless, we were able to show that a slight but persistent immune activation with a shift on the Th2 side via the IL-4 pathway was characteristic for the group of PI-IBS patients.

Additionally, in the colon mucosae from our observation group, cytokines (such as IFNγ, together with IL-1β) directly affecting epithelial transport (such as via ENaC) or barrier function (such as via occludin, tricellulin or claudins), were not present to such an extent that they had an effect on the function of the colon, which can be deduced from our Ussing experiments on the biopsies. However, we cannot rule out some contribution of the low-grade inflammation and subsequent barrier dysfunction to diarrhea in the PI-IBS patients. As a new finding in PI-IBS, we were able to show the activation of IL-22 in our cohort of patients after *C. jejuni* infection. Remarkably, IL-22 was also shown to be induced in human ex vivo colon samples after incubation with *C. jejuni* [31]. Therefore, the role of this interesting pro-inflammatory cytokine in the low-grade inflammatory response in PI-IBS merits further detailed investigation, especially because IL-22 activation is known to play a pivotal role in Crohn’s disease [32].

### 4.4. IBS, a Common Consequence from Campylobacteriosis with No Persistence of Campylobacter jejuni in the Microbiota of PI-IBS Patients

The molecular mimicry of *C. jejuni* surface antigens is reported to cause Guillain–Barré syndrome, a complication of campylobacteriosis with the induction of autoantibodies that destroy host neuronal myelin in response to *Campylobacter* LOS, resulting in demyelinating polyneuropathy [33]. Additionally, in IBS, it is assumed that infection with *C. jejuni* can trigger autoantibody formation, e.g., against vinculin, with direct effects on the intestine such as diarrhea due to destabilized TJs, as shown in a PI-IBS rat model [34,35].

Another mechanism in PI-IBS may involve histamine release from mast cells, which are located near nerve endings [36] and were not studied in our cohort. The release of histamine or PGE2 and the low-grade pro-inflammatory response can produce visceral hypersensitivity and barrier disturbance (leaky gut). These types of mediators could also induce hyperalgesia, a key symptom in IBS that deserves attention in future research in the PI-IBS entity.

As some pathogens can permanently colonize the intestine after infection, such as *Salmonella* species or *Giardia lamblia*, we verified whether *C. jejuni* could become part of the human microbiome, as observed in pigs and birds. However, detection by means of PCR analysis in our study failed to find evidence of such a permanent colonization in our PI-IBS patients, while *C. jejuni* could be detected during acute infection using this method.

### 4.5. Therapeutic Approaches in PI-IBS

Many phytopharmacological approaches have been shown to protect intestinal barrier function and to counteract inflammatory cytokine effects. Some of these have also been shown to be effective in IBS. They did not show an anti-microbial effect, but rather an immunomodulatory and barrier-stabilizing effect, such as the polyphenols curcumin or resveratrol or the hormone vitamin D (calcitriol) [6,7,8]. In addition, vitamin D has been reported to protect against macromolecule influx into *C. jejuni*-infected intestinal epithelial cells and against bacterial translocation [8]. However, their exact molecular influence on transcytotic processes in the intestinal epithelium has not yet been investigated. Furthermore, their role in the therapy of PI-IBS patients is not clear. Future research is urgently needed to evaluate such compounds in IBS that have a barrier protective function on the intestinal epithelium.

## Figures and Tables

**Table 1:** EEA1 and cytokine intensity grade (0–4) at surface cells in the colon mucosa of PI-IBS patients and controls.

Marker	Control (Mean ± SD)	PI-IBS (Mean ± SD)	*p* Value
EEA1	1.87 ± 0.20	3.57 ± 0.27	0.0005 ***
IL-1β	1.57 ± 0.24	2.90 ± 0.40	0.0168 *
IL-4	2.90 ± 0.29	3.83 ± 0.13	0.0152 *
IL-6	1.10 ± 0.07	1.10 ± 0.04	0.9999 ^ns^
IL-10	2.43 ± 0.36	1.97 ± 0.47	0.4444 ^ns^
IL-22	1.63 ± 0.26	2.80 ± 0.33	0.0190 *
INFγ	3.03 ± 0.32	2.93 ± 0.31	0.8265 ^ns^
TNFα	1.43 ± 0.16	1.33 ± 0.11	0.6171 ^ns^

The intensity of the marker signals (red-colored) at surface enterocytes was weighted as weak = 1, moderate = 2, intense = 3 or massive = 4. Evaluation was carried out with n = 6 patients in each group and 5 observations for each patient in non-overlapping high-power fields using a 40× objective. ns = not significant, * *p* < 0.05, *** *p* < 0.001, Student’s *t*-test.

## Data Availability

Not applicable.
